# The Role of the Lymph Node Ratio in Advanced Gastric Cancer After Neoadjuvant Chemotherapy

**DOI:** 10.3390/cancers11121914

**Published:** 2019-12-01

**Authors:** Karol Rawicz-Pruszyński, Bogumiła Ciseł, Radosław Mlak, Jerzy Mielko, Magdalena Skórzewska, Magdalena Kwietniewska, Agnieszka Pikuła, Katarzyna Gęca, Katarzyna Sędłak, Andrzej Kurylcio, Wojciech P. Polkowski

**Affiliations:** 1 Department of Surgical Oncology, Medical University of Lublin, 20-080 Lublin, Poland; bogumilacisel@uml.edu.pl (B.C.); jerzymielko@uml.edu.pl (J.M.); magdalenaskorzewska@uml.edu.pl (M.S.); magdalenakwietniewska@uml.edu.pl (M.K.); agnieszkapikula@uml.edu.pl (A.P.); kasiaa.geca@gmail.com (K.G.); sedlak.katarz@gmail.com (K.S.); andrzejkurylcio@uml.edu.pl (A.K.);; 2 Department of Human Physiology, Medical University of Lublin, 20-080 Lublin, Poland; radoslawmlak@uml.edu.pl

**Keywords:** gastric cancer, lymph node ratio, neoadjuvant chemotherapy

## Abstract

The ratio of positive lymph nodes (LNs) to the total LN harvest is called the LN ratio (LNR). It is an independent prognostic factor in gastric cancer (GC). The aim of the current study was to evaluate the impact of neoadjuvant chemotherapy (NAC) on the LNR (ypLNR) in patients with advanced GC. We retrospectively analyzed the data of patients with advanced GC, who underwent gastrectomy with N1 and N2 (D2) lymphadenectomy between August 2011 and January 2019 in the Department of Surgical Oncology at the Medical University of Lublin. The exclusion criteria were a lack of preoperative NAC administration, suboptimal lymphadenectomy (<D2 and/or removal of less than 15 lymph nodes), and a lack of data on tumor regression grading (TRG) in the final pathological report. A total of 95 patients were eligible for the analysis. A positive correlation was found between the ypLNR and tumor diameter (*p* < 0.001), post treatment pathological Tumour (ypT) stage (*p* < 0.001), Laurén histological subtype (*p* = 0.0001), and the response to NAC (*p* < 0.0001). A multivariate analysis demonstrated that the ypLNR was an independent prognostic factor in patients with intestinal type GC (*p* = 0.0465) and in patients with no response to NAC (*p* = 0.0483). In the resection specimen, tumor diameter and depth of infiltration, Laurén histological subtype, and TRG may reflect the impact of NAC on LN status, as quantified by ypLNR in advanced GC.

## 1. Introduction

In 2018, gastric cancer (GC) was diagnosed in 1,000,000 patients. The cause of an estimated 783,000 deaths, it was the fifth-most frequently diagnosed cancer and the third leading cause of cancer deaths worldwide [1]. Surgery is globally accepted as the only curative treatment option. Radical surgery involves gastrectomy and adequate regional lymph node dissection [2,3,4]. The latter has been suggested as the most important surgery-dependent prognostic factor in GC [5]. According to the fourth version of the Japanese Gastric Cancer Association guidelines, D1 lymphadenectomy is defined as lymph node (LN) removal from the perigastric area (stations 1–7, N1 tier), whereas N1 and N2 (D2) dissection extends along the lymph nodes at the coeliac axis and its branches (D1 plus no. 8a, 9, 10, 11p, 11d, 12a, and N2 tier) [4]. In contrast to the Far East, in the West it is recommended that surgical treatment be preceded by neoadjuvant (perioperative) chemotherapy [2,3,6,7]. LN metastases are the only independent predictor of survival after chemotherapy and surgery [8], as reported in the analysis of pathologic tumor response and nodal status in the Perioperative Chemotherapy versus Surgery Alone for Resectable Gastroesophageal Cancer (MAGIC) trial [6]. The ratio of positive LNs to the total LN harvest is called the LN ratio (LNR). A recent meta-analysis of 27 studies confirmed that as an independent prognostic factor in GC patients, higher LNR was significantly related to shorter overall survival (OS) [9]. Although several studies investigated the effect of neoadjuvant chemotherapy (NAC) on the nodal status in GC patients [7,8,10,11,12,13] and focused on the impact of NAC on the LNR in pancreatic [14], rectal [15], and breast cancer [16] patients, to the best of our knowledge, there are no data available on the influence of NAC on LNR in GC. Therefore, the aim of the current study is to evaluate the impact of NAC on LNR (ypLNR) in patients with advanced GC.

## 2. Materials and Methods

### 2.1. Study Subjects

**Figure 1:**
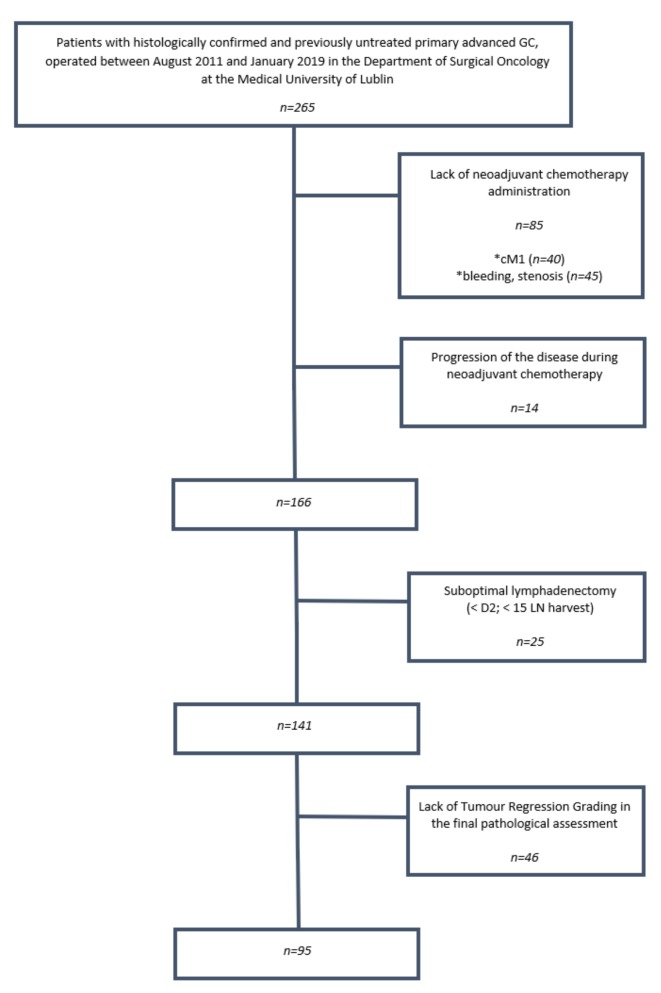
Flow chart of study inclusion and exclusion criteria.

After obtaining institutional review board approval [KE-0254/297/2018], we collected data from a prospectively maintained database of all patients with histologically confirmed and previously untreated primary advanced gastric adenocarcinoma, who were operated on between August 2011 and January 2019 in the Department of Surgical Oncology at the Medical University of Lublin (Poland). The exclusion criteria were a lack of preoperative NAC administration, suboptimal lymphadenectomy (<D2 and/or removal of less than 15 LN), and a lack of data on pathological tumor regression grading (TRG) in the final pathological report. The post treatment clinical M0/post treatment pathological M1 (ycM0/ypM1) patients were included in the study, since these patients were operated on with curative intent. The metastatic setting was revealed only after the final pathological assessment and was available after surgery. A flowchart of the inclusion and exclusion criteria of the study is shown in Figure 1. Since NAC may significantly impact the lymph node status [17], whereas inadequate lymphadenectomy (removal of <15 LNs) causes suboptimal pathological nodal (pN) staging [18], the ypLNR was not calculated in excluded patients. A total of 95 patients were eligible for analysis.

### 2.2. Preoperative Staging

Between 2011 and 2015, preoperative staging was based on computed tomography (CT) (abdominal and chest CT, and pelvic CT in females) and endoscopic ultrasonography (EUS) if there were suspicions of early GC after initial diagnostic endoscopy. Since 2016, all consecutive patients with locally advanced GC but clinically non-metastatic (cM0) GC (based on CT), have been scheduled for a staging laparoscopy with peritoneal (washings) cytology prior to evaluation of the patient on Multi Disciplinary Team (MDT) meeting.

### 2.3. Neoadjuvant (Perioperative) Chemotherapy

The perioperative epirubicin, oxaliplatin, and capecitabine (EOX) regimen consisted of 50 mg/m^2^ epirubicin and 130 mg/m^2^ oxaliplatin on day 1, with 625 mg/m^2^ capecitabine administered twice daily on days 1–21. The perioperative regimen was repeated two to three times every three weeks. The docetaxel, oxapliplatin, fluorouracil, and folinic acid (FLOT) chemotherapy consisted of oxaliplatin, 85 mg/m^2^; leucovorin, 200 mg/m^2^; and docetaxel, 50 mg/m^2^. Each was an intravenous infusion followed by fluorouracil, 2600 mg/m^2^, as a 24-h continuous intravenous infusion on day 1, repeated every two weeks. The entire cohort was scheduled for adjuvant chemotherapy; however, due to poor performance status, patient preference, and postoperative complications, 16 patients (17%) did not receive postoperative systemic treatment.

### 2.4. Tumor Regression Grading after NAC

A modified Becker’s system was used to assess TRG [19,20]—complete response/no residual tumor (Grade 1), subtotal regression/<10% residual tumor (Grade 2), partial regression/10–50% residual tumor (Grade 3), and no regression/>50% residual tumor (Grade 4). Assessment of TRG with this system is recommended by a panel of gastrointestinal pathology experts [20]. All patients were divided into two cohorts according to the TRG: patients with response to NAC (TRG = 1, 2, 3) and patients who did not respond to NAC (TRG = 4).

### 2.5. Statistical Analysis

All analyses were performed using MedCalc 15.8 (MedCalc Software, Ostend, Belgium). Data were expressed as a percentage (for categorized variable), mean, standard deviation, median, and range (for continuous variables). We considered *p* values < 0.05 as statistically significant. Spearman’s correlation test was used to calculate correlation coefficients. The comparison of ypLNR values in relation to the selected demographic and clinical variables was carried out with the use of the nonparametric U–Mann–Whitney test (the data had a non-normal distribution) and the Kruskal–Wallis test, if more than two groups were compared. Lymph node stations (LNS) were categorized into three groups: N1 tier (LNS 1–7), N2 tier (LNS 8–12a), and the complete D2 (N1 + N2) tier (LNS 1–12a). In each group, the ypLNR (the ratio of postneoadjuvant, metastatic LNs to the total LN harvest in the postoperative pathological report) was calculated for every patient. Overall survival (OS) time was defined as the length of time from the date of surgery to the patient’s death by any cause (complete data) or to the last known observation (censored data). A univariate OS analysis was performed with the use of the Kaplan–Meier estimation method (log-rank), whereas Cox logistic regression models were used in the multivariate OS analysis, with statistically significant factors from the univariate analysis (α < 0.05) included as variables. A total of 92 patients (96.8%) were included in the OS analysis. Three patients (3.2%) were lost from follow-up. 

### 2.6. Follow-Up

Initially, patients were seen in the outpatient clinic three weeks after the surgery, then every three months during the first postoperative year, every six months during the second postoperative year, and once a year thereafter. A CT scan and gastroscopy were performed 12 months after surgery, unless patients were symptomatic and/or had signs of recurrence.

## 3. Results

Among the 95 patients included in the study, 54 were males (56.8%) and 41 were females (43.2%), with the median age being 57 years. The median tumor size tumor upon pathological evaluation was 3.5 cm, and the majority of tumors were poorly differentiated (G3; 82.7%). There were 45 (47.4%) intestinal, 29 (30.5%) diffuse, and 21 (22.1%) mixed tumors. There were 44 patients (46.4%) who did not have tumor regression (TRG 4), 32 patients (33.7%) who presented with partial regression (TRG 3), 9 patients (9.4%) who presented with subtotal regression (TRG 2), and 10 patients (10.5%) who had complete response to NAC (TRG 1). There were 63 tumors (66.6%) that were at least ypT3. Additionally, 53 patients (55.8%) had lymph node metastases (N+) and 19 patients (20%) had distant metastases (ypM1) in the final pathological evaluation. The median LN harvest was 28. The clinicopathological features of selected patients are shown in Table 1.

### 3.1. ypLNR in Selected Subgroups

The median ypLNR for the entire cohort was 0.07. In patients with a tumor diameter of <3.5 cm, the median ypLNR was significantly lower than in the patients with larger (≥3.5 cm) tumors in N1 and N2, as well as in the combined N1 + N2 tiers (*p* = 0.0003, *p* = 0.009, and *p* = 0.0005, respectively). In patients with intestinal-type GC, the median ypLNR was significantly lower than in patients with diffuse- and mixed-type GC in the N1, N2, and N1 + N2 tiers (*p* = 0.0005, *p* = 0.001, and *p* = 0.0005, respectively). In patients with response to NAC, the median ypLNR was significantly lower than in patients without a NAC response in the N1, N2, and N1 + N2 tiers (*p* < 0.0001, *p* = 0.001, and *p* < 0.0001, respectively). In ypT4 patients, the median ypLNR was significantly higher than in ypT0–T3 patients in the N1 and N1 + N2 tiers (*p* = 0.001 and *p* = 0.002, respectively). With respect to nodal status, a significant difference was observed between ypN0 patients (ypLNR = 0) and ypN + patients (ypLNR >0) in the N1, N2, and D2 tiers (*p* < 0.0001). In ypM1 patients, the median ypLNR was significantly higher than in ypM0 patients in the N1, N2, and N1 + N2 tiers (*p* < 0.0001, *p* = 0.04, and *p* = 0.001, respectively). No statistically significant association was found between ypLNR and a patient’s sex, age, and tumor location and grading. Differences between ypLNR in the N1, N2, and the N1 + N2 tiers in relation to various clinicopathological features are presented in Table 2.

### 3.2. Correlation between ypLNR and Selected Clinicopathological Variables

A significant correlation was shown between the clinical Tumour (cT) stage and ypLNR in the N1 and N1 + N2 tiers (*p* = 0.0006 and *p* = 0.0024, respectively), whereas the correlation between the cT stage and ypLNR in N2 tier was nearly significant (*p* = 0.06). The maximal tumor diameter and ypLNR showed a positive correlation in the N1 and N1 + N2 tiers (*p* < 0.0001 and *p* < 0.0001, respectively). A positive correlation was found between ypLNR and the Laurén histological subtype in the N1 and N1 + N2 tiers (both *p* = 0.0001). There was an upward trend in ypLNR value in intestinal-, mixed-, and diffuse-type GC, respectively. A positive correlation was found between ypLNR and response to NAC in the N1, N2, and N1 + N2 tiers (*p* < 0.0001, *p* = 0.0009, and *p* < 0.0001, respectively). Positive correlation was also observed between ypLNR and ypT in the N1 and N1 + N2 tiers (both *p* < 0.0001) and ypLNR and ypM in the N1, N2, and N1 + N2 tiers (*p* < 0.0001, *p* = 0.03, and *p* = 0.001, respectively). A positive correlation was found between ypLNR and ypN in the N1, N2, and N1 + N2 tiers (*p* < 0.0001, *p* < 0.0001, and *p* < 0.000, respectively). Results of the Spearman’s rank correlation coefficient between ypLNR and selected clinicopathological variables are shown in Table 3.

### 3.3. Tumor Survival Analysis

In the univariate analysis of OS, ypLNR > median showed prognostic significance in patients with intestinal-type GC (11 vs. 37 months, *p* = 0.0114) and diffuse-type GC (15 vs. 39 months, *p* = 0.0008), as well as in patients with response to NAC (14 vs. 39 months; *p* = 0.0162) and in patients with no response to NAC (11 vs. 34 months; *p* = 0.0097). A multivariate analysis demonstrated that ypLNR was an independent prognostic factor in intestinal-type GC (*p* = 0.0465) and in patients with no response to NAC (TRG 4) (*p* = 0.0483). The results of the uni- and multivariate survival analysis are presented in Table 4. The median OS of patients with ypLNR ≤0.07 was 37 months, whereas in patients with ypLNR >0.07, the median OS was 11 months (*p* = 0.0002; log-rank test; HR 2.29; 95% CI: 1.36–3.84). The median follow-up for all patients, ypM0 patients and ypM1 patients was 20, 29 and 9 months, respectively. During follow-up, 71% of patients died. The date of data cut-off was 4 October, 2019.

## 4. Discussion

The current study enabled us to distinguish a ypLNR high-risk group among GC patients after NAC. Tumor diameter ≥ 3.5 cm, Laurén intestinal subtype, lack of response to NAC (TRG 4), serosal infiltration, lymph node metastases, and distant metastases were significantly associated with higher ypLNR.

The influence of NAC on nodal status in GC patients has been investigated meticulously [21]. Wu et al. [22] evaluated the influence of clinical, pathological, and treatment variables on the total LN harvest and the number of metastatic LNs after NAC in patients with GC. The study showed that NAC for GC reduced the total LN count and increased the number of patients who had <15 LN harvested. Thus, a decrease in total LN harvest should be expected in patients undergoing resection after neoadjuvant chemotherapy. In a study conducted by Ji et al. [23], the total LN harvest was an independent prognostic factor in ypN0 GC patients, with a minimum LN harvest of 22. Interestingly, in these patients, surgery alone was even more beneficial than neoadjuvant chemotherapy, as reported by Ronellenfitsch et al. [17]. However, in ypN+ patients, survival was longer in those who received NAC [17] and total LN harvest should exceed 30 in order to avoid stage migration after surgery [24].

Recent data from Asian [25,26], North American [27], and European [28,29] populations showed that LNR is considered a more accurate and reliable parameter than TNM classification in terms of GC prognosis. Additionally, LNR could be a better option to compensate for the stage migration effect. The predictive value for prognosis increases with a higher number of retrieved lymph nodes, as shown in a high-volume study from Korea [30]. Moreover, LNR is a prognostic indicator for patients who develop GC liver metastases, as well as nodal and peritoneal recurrences after radical resection [31,32].

The Laurén classification remains an important clinical factor in treatment of GC. A recent study by Wang et al. demonstrated that LNR might be used as an independent predictor of survival in patients with diffuse-type GC [33]. Jimenez et al. [34] studied the chemosensitivity of GC according to Laurén subtypes. Diffuse-type GC was found to be less chemosensitive and was associated with increased mortality. The recent study by Xu et al. [35] focused on the prognostic value of TRG in perioperative treatment of advanced GC. The Laurén classification and the ypT stage were independent factors for TRG, whereas TRG itself was a prognostic variable for ypN+ patients. In the present study, patients with response to NAC had significantly lower ypLNR when compared to nonresponders. Moreover, the Laurén histological subtype analysis revealed an upward trend in ypLNR value—the mean ypLNR was lowest in intestinal-type GC, intermediate in mixed-type GC, and highest in diffuse-type GC. These results show the potential prognostic information of ypLNR in Western patients with advanced GC by means of response to NAC in different histological subtypes.

The accurate prediction of response to neoadjuvant and adjuvant chemotherapy remains a challenge [36,37,38]. Due to histological heterogeneity, tumor behavior throughout the clinical management of GC remains uncertain. Improved understanding of GC biology will successively favor tailored surgery. Further research could possibly introduce LNR as a new biomarker [39], since it is closely associated with epidermal growth factor receptor (EGFR) expression [40].

In the era of NAC in GC, the potential effect of systemic treatment on lymph node involvement should be investigated. LNR proved to be an important prognostic factor in the adjuvant setting.

This study contains certain limitations. Due to its retrospective nature, it cannot identify causation. Due to the relatively small sample size, a subgroup stratification analysis might be biased. Moreover, our pathological evaluation did not include assessment of molecular subtype, tumor budding, and lymph node regression, which could be of potential prognostic significance in this setting.

## 5. Conclusions

In resection specimens, tumor diameter and depth of infiltration, Laurén histological subtype, and TRG may reflect the impact of NAC on LN status, as quantified by ypLNR in advanced GC. When validated in prospective studies, ypLNR could serve as a simple and objective parameter in the clinical evaluation of NAC.

## Figures and Tables

**Table 1:** Clinicopathological variables.

Variables	No. of Patients *n* = 95 (%)
Sex	
Male	54 (56.8%)
Female	41 (43.2%)
Age (years)	
Average	57.37
Standard deviation (±)	10.90
Median (min-max)	57 (31–77)
Tumor maximal diameter (cm)	
Average	4.2
Standard deviation (±)	2.7
Median (min-max)	3.5 (1–15)
Tumor location	
Upper 1/3	29 (31%)
Middle 1/3	27 (28%)
Distal 1/3	39 (41%)
Tumor depth	
Mucosa	2 (2%)
Submucosa	7 (7%)
Muscularis Propria	35 (37%)
Subserosa	14 (15%)
Serosa	37 (39%)
Lauren histological subtype	
Intestinal	45 (47.4%)
Diffuse	29 (30.5%)
Mixed	21 (22.1%)
Grading	
G1	6 (8%)
G2	27 (9.3%)
G3	62 (82.7%)
No. of NAC cycles	
1	2 (2%)
2	8 (8%)
3	56 (60%)
4	20 (21%)
6	6 (6.38%)
8	2 (2.13%)
NAC regimen	
EOX	83 (87.2%)
FLOT	12 (12.8%)
Tumor regression grading (TRG) (Classification of response)	
Complete (Grade 1)	10 (10.5%)
Subtotal (Grade 2)	9 (9.4%)
Partial (Grade 3)	32 (33.7%)
Minimal/No regression (Grade 4)	44 (46.4%)
ypT	
T0	6 (6.3%)
T1	6 (6.3%)
T2	20 (21%)
T3	40 (42%)
T4	23 (24.6%)
ypN	
N0	42 (44.2%)
N1	7 (7.4%)
N2	14 (14.4%)
N3	32 (34%)
ypM	
M0	76 (80%)
M1	19 (20%)
No. of examined lymph nodes	
Mean	32
Standard deviation (±)	14
Median (min-max)	28 (16–81)

NAC: neoadjuvant chemotherapy. EOX: epirubicin, oxaliplatin and capecitabine. FLOT: docetaxel, oxapliplatin, fluorouracil and folinic acid.

**Table 2:** ypLNR in selected clinicopathological variables in the N1, N2, and N1 + N2 (D2) tiers.

Variable:	N1		N2		N1 + N2 (D2)	
	Me	*p*	Me	*p*	Me	*p*
Sex ^¥^						
Male	0.08	0.64	0.00	0.56	0.00	0.41
Female	0.00		0.00		0.07	
Age (years) ^¥^						
<57	0.08	0.54	0.00	0.83	0.09	0.40
≥57	0.02		0.00		0.04	
Maximal tumor dimension (cm) ^¥^						
<3.5	0.00	0.0003	0.00	0.009	0.00	0.0005
≥3.5 cm	0.38		0.00		0.31	
Tumor location ^#^						
Upper 1/3	0.00	0.09	0.00	0.28	0.02	0.09
Middle 1/3	0.30		0.00		0.06	
Lower 1/3	0.03		0.00		0.22	
Laurén histological subtype ^#^						
Intestinal	0.00	0.0005	0.00	0.001	0.00	0.0005
Diffuse	0.45		0.00		0.30	
Mixed	0.30		0.14		0.22	
Grading ^#^						
G1	0.01	0.27	0.00	0.73	0.01	0.46
G2	0.00		0.00		0.07	
G3	0.10		0.00		0.09	
Response to NAC (TRG)^¥^						
Response to NAC (TRG 1–3)	0.00	<0.0001	0.00	0.0011	0.00	<0.0001
No response to NAC (TRG 4)	0.40		0.07		0.30	
ypT ^¥^						
ypT0-T3	0.00	0.001	0.00	0.06	0.00	0.002
ypT4	0.50		0.08		0.31	
ypN ^¥^						
N0	0.00	<0.0001	0.00	<0.0001	0.00	<0.0001
N1-N3b	0.48		0.24		0.36	
ypM ^¥^						
M0	0.00	0.0001	0.00	0.04	0.00	0.001
M1	0.53		0.08		0.30	

Me: median. TRG: tumor regression grading; ^¥^ U–Mann–Whitney test, ^#^ Kruskal–Wallis test.

**Table 3:** Spearman’s rank correlation coefficient between ypLNR and selected clinicopathological variables.

Variable (*n* = 95)	ypLNR					
	N1		N2		N1 + N2 (D2)	
	R (Spearman)	*p*	R (Spearman)	*p*	R (Spearman)	*p*
Age	−0.015	0.88	0.005	0.96	−0.024	0.82
cT	0.344	0.0006	0.192	0.06	0.308	0.002
Tumor max. diameter	0.455	<0.0001	0.246	0.01	0.420	<0.0001
Grading	0.166	0.10	0.068	0.51	0.126	0.22
Laurén histological subtype	0.399	0.0001	0.0179	0.8632	0.387	0.0001
Response to NAC (TRG)	0.528	<0.0001	0.335	0.0009	0.503	<0.0001
No. of NAC cycles	0.120	0.24	0.187	0.07	0.151	0.14
ypT	0.436	<0.0001	0.270	0.008	0.422	<0.0001
ypN	0.903	<0.0001	0.744	<0.0001	0.953	<0.0001
ypM	0.405	<0.0001	0.213	0.03	0.330	0.001

**Table 4:** The effect of ypLNR on overall survival (OS) based on the Laurén classification and TRG.

Variable	Univariate		Multivariate
	Months	HR (95%CI) *p*	HR (95%CI) *p*
Intestinal-type GC			
ypLNR > median (0.00)	11	2.69 (1.09–6.64)	2.87 (1.02–8.06) *
ypLNR ≤ median (0.00)	37	0.0114	0.0465
Diffuse-type GC			
ypLNR > median (0.30)	15	2.99 (1.18–7.60)	2.28 (0.60–8.47)
ypLNR ≤ median (0.30)	39	0.0008	0.3488
Mixed-type GC			
ypLNR > median (0.22)	10	1.13 (0.41–3.14)	0.48 (0.07–3.17)
ypLNR ≤ median (0.22)	15	0.8150	0.4453
Response to NAC (TRG 1–3)			
ypLNR > median (0.00)	14	2.18 (0.97–4.91)	2.38 (0.94–6.03)
ypLNR ≤ median (0.00)	39	0.0162	0.0683
No response to NAC (TRG 4)			
ypLNR > median (0.30)	11	2.29 (1.15–4.55)	2.46 (1.01–5.99) **
ypLNR ≤ median (0.30)	34	0.0097	0.0483

Tumor* grading, tumor maximal diameter, ypM, and ypT were significant variables in univariate analysis. ** grading, tumor location, ypM, and ypT were significant variables in univariate analysis.
